# Integrin α9β1 deficiency does not impact the development of atherosclerosis in mice

**DOI:** 10.1016/j.heliyon.2024.e25760

**Published:** 2024-02-09

**Authors:** In-Hyuk Jung, Nathan O. Stitziel

**Affiliations:** a Center for Cardiovascular Research, Division of Cardiology, Department of Medicine, Washington University School of Medicine, Saint Louis, MO, 63110, USA; b Department of Genetics, Washington University School of Medicine, Saint Louis, MO, 63110, USA

**Keywords:** SVEP1, Integrin α9β1, Extracellular matrix, Atherosclerosis, Mouse models of human disease

## Abstract

Sushi, von Willebrand factor type A, EGF and pentraxin domain containing 1 (SVEP1) is an extracellular matrix protein that causally promotes cardiovascular disease in humans and mice. However, the receptor mediating the effect of SVEP1 on the development of disease remains unclear. We previously demonstrated that depleting either vascular smooth muscle cell (VSMC)- or myeloid cell-derived integrin α9β1, the first receptor that was identified to interact with SVEP1, did not phenocopy the disease-abrogating effect of depleting SVEP1. Due to its wide expression in tissues and cell types, here we extend this line of investigation to definitively determine if integrin α9β1 impacts the development of atherosclerosis. In a mouse model of atherosclerosis, we found that depleting integrin α9β1 in all cells did not alter plaque size or characteristics of plaque complexity when compared to wild type mice. Further, the significant SVEP1-mediated effects on increase in macrophage content and VSMC proliferation within the atherosclerotic plaque were not altered in animals lacking integrin α9β1. Together, these findings strongly suggest that integrin α9β1 is not responsible for mediating the SVEP1-induced promotion of atherosclerosis and support further studies aimed at characterizing other receptors whose interaction with SVEP1 may represent a therapeutically targetable interaction.

## 1 Introduction

Atherosclerotic cardiovascular diseases including coronary artery disease (CAD) are leading causes of morbidity and mortality worldwide [[1], [2], [3], [4]], highlighting the need for a better understanding of the pathological mechanisms underlying CAD with the potential to identify novel therapeutic targets for the prevention and treatment of disease. Previously, we found that a highly conserved missense polymorphism in *SVEP1* which encodes Sushi, von Willebrand factor type A, EGF pentraxin domain-containing 1 (SVEP1), a secreted extracellular matrix (ECM) protein, associated with an increased risk of disease in humans [5]. Using complimentary mouse models and human genetics, we confirmed that SVEP1 promoted the development of atherosclerosis and increased plaque complexity [6]. Within the arterial wall, VSMC-derived SVEP1 was found to significantly promote VSMC proliferation in the developing plaque. Further, both whole body and VSMC-specific depletion of SVEP1 reduced the recruitment of monocytes into the plaque lesion within the arterial wall, resulting in lower plaque macrophage content. Despite confirmation that SVEP1 was causally related to the development of atherosclerosis, the cellular receptor responsible for mediating the effect of SVEP1 remained unclear.

The first receptor shown to directly interact with SVEP1 (and thus a candidate for mediating its effect on disease) was integrin α9β1 [7]. The integrin superfamily of proteins are transmembrane heterodimeric receptors consisting of α and β subunits which can transduce signals in response to a range of diverse extracellular ligands [8,9]. Their role is mainly focused on the adhesive interactions or migration of cells by binding to ECM proteins, cell-surface ligands, and other soluble ligands [10]. Integrin α9β1 is involved in lymphangiogenesis [11] and has been implicated in VSMC phenotype switching [[12], [13], [14]] along with VSMC proliferation and migration [12]. Other *in vitro* results suggest SVEP1 may regulate vascular contraction through integrins α9 and α4 [15]; however, *in vivo* physiology studies failed to demonstrate a significant difference in vascular reactivity for mice lacking SVEP1 [16]. Neutrophil specific integrin α9β1 depletion appears to improve phenotypes in stroke models by limiting cerebral thrombosis and inflammation [17]. In other disease settings, integrin α9β1 has been reported to influence tumor cell migration, metastatic invasion, epithelial-mesenchymal transition, and acute aortic dissection [13,[18], [19], [20]].

Based on the known interaction between SVEP1 and integrin α9β1 along with the expression of integrin α9β1 by VSMCs and myeloid cells including monocytes and macrophages, we asked if integrin α9β1 was responsible for mediating either the cell autonomous or cell non-autonomous effects of SVEP1 in the pathogenesis of atherosclerosis [21]. Using mouse models of *Itga9* depletion in either VSMCs or myeloid cells, we did not observe any differences in the size or complexity of atherosclerotic plaques in either model, strongly suggesting that integrin α9β1 is unlikely to be the receptor mediating the effects of SVEP1 in these cells.

Despite this result, integrin α9β1 is also expressed by other tissues and cells including endothelial cells [22,23] which are known to play an important role in the development of atherosclerosis [[24], [25], [26]]. As a result, we sought to definitively determine if integrin α9β1 has a potential role in the development of atherosclerosis by testing whether depleting integrin α9β1 in all cells would influence plaque development. To address this question, we induced hypercholesterolemia in animals with or without whole-body depletion of integrin α9β1 followed by feeding high fat diet (HFD) and investigated the role of integrin α9β1 in atherosclerotic plaque development.

## 2 Methods

### 2.1 Mouse strains and induction of *Itga9* knockout by Cre recombinase

Mice harboring *loxP* sites flanking a critical exon of *Itga9* (*Itga9*^*flox/flox*^, hereafter referred to as *Itga9*^*fl/fl*^ mice) [27] were a gift from D. Sheppard (University of California San Francisco, United States) and L. Van De Water (Albany Medical College, United States). We crossed *Itga9*^*fl/fl*^ mice with mice expressing a tamoxifen-inducible Cre recombinase (*Rosa26-CreER*^*T2*^, #008463, the Jackson Laboratory, United States) to generate *Itga9*^*fl/fl*^*Rosa26-CreER*^*T2*^ mice (hereafter referred to as *Itga9*^*KO*^). We maintained *Itga9*^*fl/+*^*Rosa26-CreER*^*T2*^ animals (hereafter referred to as *Itga9*^*het*^) as breeders to generate experimental *Itga9*^*fl/fl*^*Rosa26-CreER*^*T2*^ (*Itga9*^*KO*^) and control *Rosa26-CreER*^*T2*^ (*Itga9*^*WT*^) littermates. To activate Cre-recombinase, 2 mg tamoxifen (#T5648, Sigma-Aldrich, United States) in 0.1 ml of corn oil (#C8267, Sigma-Aldrich, United States) was administered by oral gavage for 5 consecutive days (total 10 mg per mouse) to all experimental and control animals starting at 6 weeks of age, followed by a tamoxifen wash out period of 1.5 weeks. All mice were housed in a pathogen-free environment at the Washington University School of Medicine animal facility and maintained on a 12-h light/12-h dark cycle with a room temperature of 22° ± 1 °C. All experiments and procedures were approved by the Washington University School of Medicine Animal Studies Committee.

### 2.2 Induction and assessment of atherosclerosis

To induce hypercholesterolemia, 8-week-old-tamoxifen oral gavaged mice were intravenously injected with 5 × 10^11^ vector genome copies of AAV8-D377Y-mPCSK9 (Vector Biolabs, United States) and immediately placed on a HFD containing 21% fat by weight (42% kcal from fat) and 0.2% cholesterol (#TD88137, Envigo Teklad, United States). Mouse plasma samples were prepared from the collected blood through retro-orbital plexus at baseline (0), 2, 8, and 16 weeks after virus injection, and total cholesterol concentrations were measured using the kit (#C7510, Pointe Scientific, United stated). Detailed tissue processing including plasma, aortic roots, and whole arteries from the aortic arch to the iliac for *en face* analysis were described previously [6]. Briefly, isolated aortas were opened longitudinally, following fixation with 4% paraformaldehyde (PFA) at 4 °C overnight. Aortas were then stained with 0.5% Oil Red O in propylene glycol (#O1516, Sigma-Aldrich, United States), followed by de-staining with 85% propylene glycol to reduce background staining. For analysis of plaque in the aortic root, isolated hearts were fixed with 4% PFA at 4 °C overnight, and embedded into optimal cutting temperature (OCT) compound (#4583, Sakura Finetek, Japan). Tissues were cut with 5-μm-thickness, and further processed for Oil Red O staining. Measurement of plaque was performed using 6-8 sections per artery to get the average value of size. The atherosclerotic plaque area was digitized and calculated using AxioVison (Carl Zeiss, Germany).

### 2.3 Immunohistochemistry and immunofluorescent staining

4% PFA-fixed frozen aortic root sections with 5-μm-thickness were used for all studies. To assess plaque complexity, necrotic core and collagen content were visualized by hematoxylin and eosin (H&E, #HHS80 and #HT110180) and Masson-Goldner's trichrome staining (#100485, all purchased from Sigma-Aldrich, United States), which were performed according to the manufacturer's protocols. For immunofluorescent staining, slides were permeabilized with 0.5% tritonX-100 for 10 min, followed by blocking with 5% donkey serum (#D9663, Sigma-Aldrich, United States) in 0.5% tritonX-100 for 30 min at room temperature. Anti-Itga9 (#AF3827, 1:40, R&D systems, United States) was used to detect Itga9 expression, and normal goat IgG (#NI02-100UG, 1:40, EMD Millipore, United States) was used as isotype control. Both antibodies were then visualized with donkey anti-goat Alexa594 (#A11058, 1:400, Invitrogen, United States). Anti-Mac3 (#550292, 1:100, BD Biosciences, United States), anti-SMα-actin-cy3 (#C6198, 1:500, Sigma-Aldrich, United States) were used, and then visualized with donkey anti-rat Alexa488 (#A21208, 1:400, Invitrogen, United States) was used. Nuclear counterstaining and slide mounting were performed with Prolong Diamond Antifade with DAPI (#P36862, Invitrogen, United States).

### 2.4 Immunoblot assay

Immunoblots were performed as briefly follows. Quantification of protein content was determined using a bicinchoninic acid assay (#23225, Pierce BCA Protein Assay Kit, United States). Tissue lysates were then reduced with dithiothreitol in lithium dodecyl sulfate sample buffer (#NP0007, Invitrogen, United States). Equal protein amounts were added to 4–20% gradient polyacrylamide gels (#4561094, BioRad, United States) and electrophoresed prior to transferring to a polyvinylidene fluoride membrane (#1620260, BioRad, United States). Membranes were blocked in 5% BSA/Tris-Buffered Saline with tween 20 for 30 min. Anti-Itga9 (#AF3827, 1:1000, R&D systems, United States) was used to detect Itga9 expression, and IRDye 680RD donkey anti-goat IgG (#926–68074, 1:5000, LI-COR, United States) was used as its secondary antibody. Anti-tubulin (#12004166, 1:3000, Biorad, United States) was used as loading control.

### 2.5 Measurement of cell proliferation

Mice were injected intraperitoneally with 50 mg kg^−1^ of 5-Ethynyl-2′-deoxyuridine (EdU) (#61135-33-9, Cayman Chemicals, United States) in 100 μL volume 96 h before sacrifice over 16 weeks of HFD feeding. 4% PFA-fixed frozen aortic root sections with 5-μm-thickness were used, and EdU staining was performed using a Click & Go Cell reaction buffer kit with Alexa-Fluor 488 (#1263, Click chemistry tools, United States) following the manufacturer's instructions. Anti-Mac3 and anti-SMα-actin-cy3 were used to visualize the distribution of each cell type. Quantification of proliferation was performed by counting the numbers of EdU-positive nuclei in plaque area.

### 2.6 Statistical analysis

For all animal model data, one-way analysis of variance (ANOVA), or unpaired nonparametric Mann-Whitney test were used. Statistical analyses were performed with GraphPad Prism software version 10.0 (GraphPad Software, United States).

## 3 Results

### 3.1 Whole body depletion of integrin α9β1 does not regulate plaque development or its complexity

**Fig. 1:**
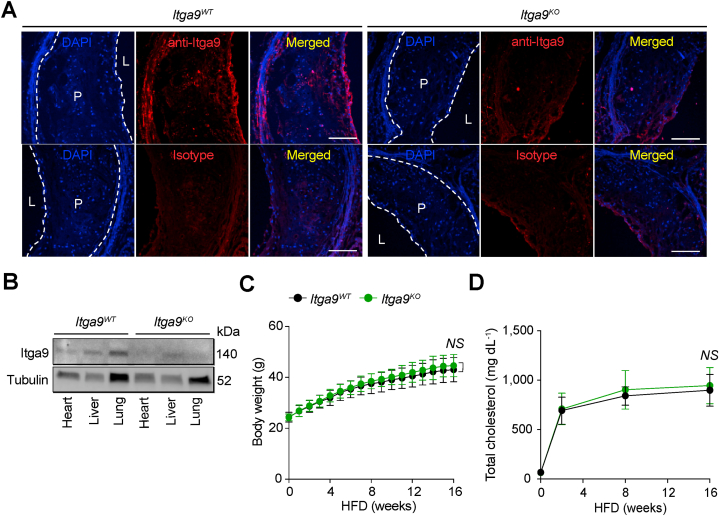
**Itga9 expression in aortic root and plasma lipids during HFD feeding.***Itga9*^*WT*^ and *Itga9*^*KO*^ mice were gavaged with 100 μL of 20 mg ml^−1^ of tamoxifen at 6 weeks of age for 5 consecutive days. Hypercholesterolemia was induced by i.v. injection of AAV8-PCSK9 at 8 weeks of age, followed by HFD feeding for 16 weeks. **(A)** Representative images of Itga9 immunofluorescence (red) in plaque burden of aortic root from *Itga9*^*WT*^ (left panel) and *Itga9*^*KO*^ mice (right panel) to validate Itga9 depletion. In lower panels for both genotypes, isotype control staining using goat IgG was performed to check antibody specificity. Nuclei were counterstained with DAPI (blue). **(B)** Immunoblots of Itga9 in tissues including heart, liver, and lung isolated from *Itga9*^*WT*^ and *Itga9*^*KO*^ mice to validate Itga9 depletion. Tubulin was used as loading control. The uncropped versions of these blots are provided in Supplementary Fig. 1. **(C)** Weekly body weight of *Itga9*^*WT*^ and *Itga9*^*KO*^ mice over the HFD feeding period. **(D)** Total cholesterol (at 0, 2, 8, and 16 weeks) over the HFD feeding. *n* = 17 for *Itga9*^*WT*^ and *n* = 11 for *Itga9*^*KO*^ in **(C)***, n* = 18 for *Itga9*^*WT*^ and *n* = 12 for *Itga9*^*KO*^ in **(D)**. Data are shown as mean ± SD and analyzed with two-way ANOVA with repeated measures **(C and D)**. NS, not significant. Scale bars, 100 μm. L, lumen; P, plaque.

**Fig. 2:**
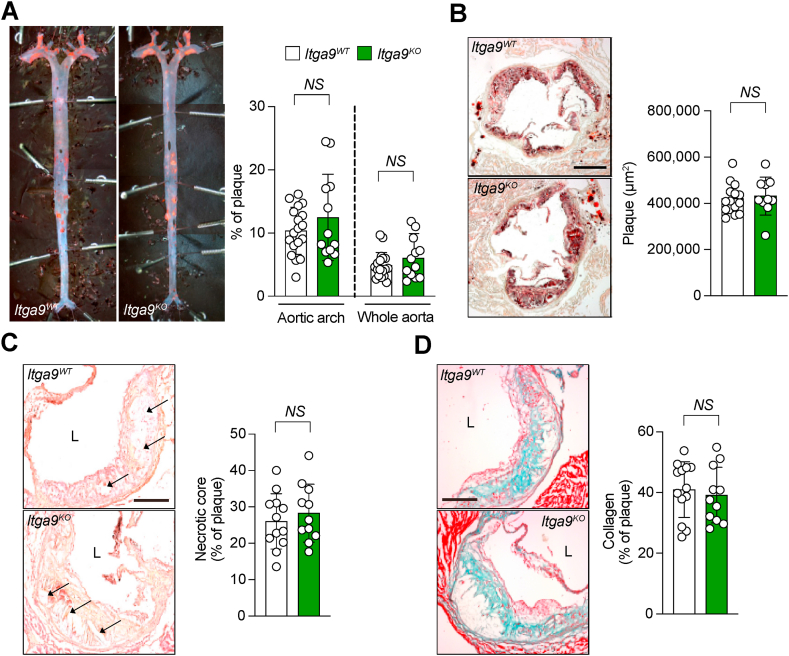
**Whole body depletion of Itga9 does not impact atherosclerotic plaque formation or its complexity. (A)***En face* Oil Red O–stained mouse aortas. Quantification of Oil Red O–stained plaque area as a percentage of the aortic arch and entire aortic area. **(B)** Oil Red O–stained aortic root cross sections. Quantification of Oil Red O–stained plaque area. **(C)** Necrotic core of aortic roots outlined on hematoxylin and eosin–stained sections. Quantification of necrotic core as a percentage of plaque area. **(D)** Collagen staining of aortic roots using Masson-Goldner trichrome method. Quantification of collagen as a percentage of plaque area. Scale bars, 500 μm **(B)**, and 200 μm **(C and D)**. *n* = 18 for *Itga9*^*WT*^ and *n* = 12 for *Itga9*^*KO*^ in **(A)**, *n* = 14 for *Itga9*^*WT*^ and *n* = 10 for *Itga9*^*KO*^ in **(B)**, *n* = 12 for *Itga9*^*WT*^ and *n* = 11 for *Itga9*^*KO*^ in **(C)**, *n* = 13 for *Itga9*^*WT*^ and *n* = 11 for *Itga9*^*KO*^ in **(D)**. Data are shown as mean ± SD and analyzed with unpaired nonparametric Mann-Whitney test **(A through D)**. NS, not significant. L, lumen.

Since integrin α9β1 is required for normal embryonic development [28], we generated mice with a whole body inducible depletion of integrin α9β1 (*Itga9*^*fl/fl*^*Rosa26-CreER*^*T2*^, hereafter referred to as *Itga9*^*KO*^) along with wild type control *Rosa26-CreER*^*T2*^ (hereafter referred to as *Itga9*^*WT*^) mice to determine whether integrin α9β1 has a significant regulatory role in the development of atherosclerotic plaque. First, we validated our model of integrin α9β1 depletion by using immunofluorescence staining in the aortic root, finding that *Itga9*^*KO*^ mice had a substantial reduction of Itga9 expression compared to *Itga9*^*WT*^ control mice (Fig. 1A). We also confirmed Itga9 depletion using protein immunoblotting in heart, liver, and lung tissues isolated from *Itga9*^*KO*^ and *Itga9*^*WT*^ mice (Fig. 1B). We then determined whether integrin α9β1 depletion modulated the development of atherosclerotic plaque by inducing hypercholesterolemia with intravenous injection of AAV8-PCSK9 to *Itga9*^*WT*^ and *Itga9*^*KO*^ mice, followed by feeding a HFD for 16 weeks. We found that there were no differences in body weight (Fig. 1C) and total cholesterol (Fig. 1D) between genotype groups. We were unable to detect any difference in the size of atherosclerotic plaque in either the aortic arch or the whole aorta by *en face* preparation (Fig. 2A) as well as aortic root regions (Fig. 2B) between experimental groups. Additionally, atherosclerotic plaques from *Itga9*^*KO*^ mice did not show any differences in characteristics of plaque complexity including necrotic core area (Fig. 2C) and collagen content (Fig. 2D) compared to *Itga9*^*WT*^ control mice. Together, these data suggest that integrin α9β1 does not substantially contribute to atherosclerotic plaque development.

### 3.2 Whole body depletion of integrin α9β1 does not induce proliferation of macrophages and VSMCs

**Fig. 3:**
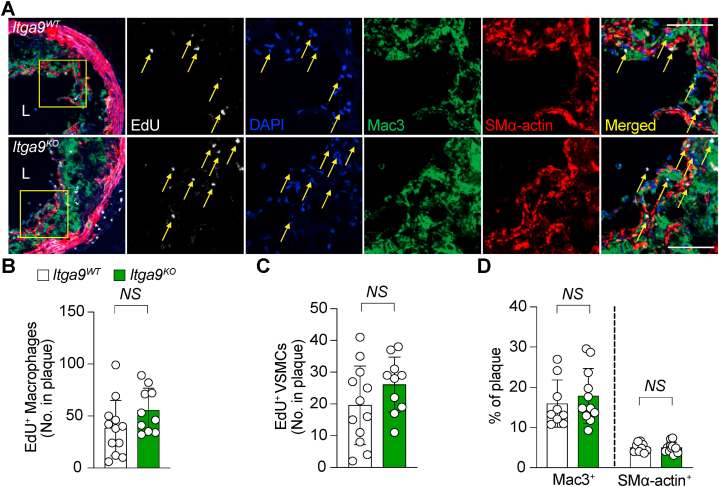
**Whole body depletion of Itga9 does not alter the proliferation of macrophages or VSMCs in atherosclerotic plaques.** To determine proliferation of cells *in vivo*, EdU (5-ethynyl-2′-deoxyuridine) was administered intraperitoneally to *Itga9*^*WT*^ and *Itga9*^*KO*^ mice 96 h before sacrifice after 16-weeks of HFD feeding. Click assay was performed to visualize EdU incorporation. **(A)** Representative images of EdU incorporated proliferating Mac3-positive (macrophages) and SMα-actin-positive (VSMCs) cells within plaque lesion. Outlined areas (yellow box) indicate the regions magnified in subsequent panels. Scale bars, 50 μm. **(B)** Quantification of EdU^+^Mac3^+^ cells. **(C)** Quantification of EdU^+^SMα-actin^+^ cells. **(D)** Quantification of Mac3^+^ and SMα-actin ^+^ cells as a percentage of plaque area. *n* = 12 for *Itga9*^*WT*^ and *n* = 10 for *Itga9*^*KO*^ in **(B and C)**, *n* = 9 for *Itga9*^*WT*^ and *n* = 11 for *Itga9*^*KO*^ in **(D)**. Data are shown as mean ± SD and analyzed with unpaired nonparametric Mann-Whitney test **(B through D)**. NS, not significant. L, lumen.

The extracellular matrix (ECM) plays a critical role in orchestrating cellular responses to

tissue injury, including promoting cell proliferation and differentiation [29,30]. As an important mechanism of atherosclerotic plaque growth, cell proliferation occurs among inflammatory cells and VSMCs [31] and our prior work found that SVEP1 both promoted VSMC proliferation and increase in macrophage content within the atherosclerotic plaque. To assess if integrin α9β1 might be mediating these SVEP1-induced effects, 5-Ethynyl-2′-deoxyuridine (EdU) was administered to mice intraperitoneally 96 h before sacrifice to label proliferating cells (Fig. 3A). We did not observe any difference in the numbers of EdU positive macrophages (Fig. 3B) or VSMCs (Fig. 3C) between genotype groups. We also did not observe any significant differences in the overall amounts of macrophages and VSMCs (Fig. 3D), both of which can be indicators of advanced plaque phenotypes, suggesting that integrin α9β1 is not likely mediating the effect of SVEP1 on these plaque characteristics.

## 4 Discussion

Based on our previous studies of integrin α9β1 on myeloid cells and VSMCs [21], here we extend this line of investigation to definitively determine if integrin α9β1 plays a significant role in the development of atherosclerosis by generating mice with an inducible depletion of integrin α9β1 in all cells. Similar to our prior study [21], we did not observe any differences in the size or complexity of atherosclerotic plaques as well as proliferation of macrophages and VSMCs between genotypes, suggesting that integrin α9β1 itself does not have a significant impact on the pathogenesis of atherosclerosis in mice and is not likely to be responsible for mediating the atherosclerotic-promoting effect of SVEP1.

Taken together, these results strongly suggest the presence of additional binding partners for SVEP1, at least one of which is responsible for mediating the effect of SVEP1 in the development of atherosclerosis. In fact, since the discovery of integrin α9β1 as the first protein known to interact with SVEP1, additional SVEP1 binding proteins have been identified. For example, the lymphangiogenic factor angiopoientin-2 (Ang-2) has been reported to bind to SVEP1, which was found to potentiate forkhead box protein c2 (FOXC2) expression in cultured lymphatic endothelial cells (LECs) [32]. Ang-2 acts as an agonist/antagonist for Tie receptors in a context-dependent manner [33] and has been shown to play a role in promoting inflammation in response to tumor necrosis factor-α (TNF-α) [34]. Interestingly, the expression of Tie receptors including Tie1 and Tie2 are lower in SVEP1 deficient mice [32] and Tie2 functions as an endothelial angiopoietin receptor that protects from atherosclerosis [35], raising the possibility that SVEP1 may modulate the Ang-Tie system in endothelial cells which could impact the development of atherosclerosis.

Our group also recently identified a high affinity, disease-relevant, and potentially targetable interaction between SVEP1 and Platelet and Endothelial Aggregation Receptor 1 (PEAR1), a receptor tyrosine kinase-like protein [16]. Similar to SVEP1, PEAR1 has EGF (epidermal growth factor) repeat-containing domains in its extracellular domain [36] and is expressed in endothelial cells, platelets, and VSMCs [16,36,37] with patterns of tissue expression that are highly correlated with SVEP1 [16]. These two genes also share concordant human disease associations including platelet reactivity [38,39] and CAD [[40], [41], [42]], suggesting their interaction may be therapeutically relevant [16].

## 5 Conclusion

In summary, our results indicate that integrin α9β1 does not appear to mediate the effect of SVEP1 in promoting atherosclerosis. Future studies will be needed to determine the alternative receptor(s) that mediate the role of SVEP1 in promoting vascular disease.

## Sources of funding

This work was supported in part by grants from the 10.13039/100000002National Institutes of Health (10.13039/100000002NIH) to NOS (R01HL159171, UM1HG008853), by the Longer Life Foundation: A RGA/Washington University Collaboration (LLF 2021-007) to NOS, and by the Foundation for Barnes-Jewish Hospital (NOS).

## Declarations

The Washington University Institutional Animal Care and Use Committee approved all animal experiments and procedures under protocol number 21–0345. IHJ and NOS are listed as co-inventors on US Patent App. 17/978,128 assigned to Washington University focused on SVEP1 and PEAR1.

## CRediT authorship contribution statement

**In-Hyuk Jung:** Writing – review & editing, Writing – original draft, Methodology, Investigation, Formal analysis, Conceptualization. **Nathan O. Stitziel:** Writing – review & editing, Writing – original draft, Supervision, Project administration, Funding acquisition, Conceptualization.

## Declaration of competing interest

The authors declare the following financial interests/personal relationships which may be considered as potential competing interests:Nathan Stitziel reports financial support was provided by 10.13039/100000002National Institutes of Health. Nathan Stitziel has patent pending to Washington University. In-Hyuk Jung has patent pending to Washington University.

## References

[1] Galkina E., Ley K. (2009). Immune and inflammatory mechanisms of atherosclerosis (*). Annu Rev Immunol.

[2] Libby P. (2021). The changing Nature of atherosclerosis: what we thought we knew, what we think we know, and what we have to learn. Eur Heart J.

[3] Libby P., Buring J.E., Badimon L., Hansson G.K., Deanfield J., Bittencourt M.S., Tokgözoğlu L., Lewis E.F. (2019). Atherosclerosis. Nat Rev Dis Primers.

[4] Ross R. (1996). Genetically modified mice as models of transplant atherosclerosis. Nat Med.

[5] Stitziel N.O., Stirrups K.E., Masca N.G., Erdmann J., Ferrario P.G., König I.R., Weeke P.E., Webb T.R., Auer P.L., Schick U.M., Lu Y., Zhang H., Dube M.P., Goel A., Farrall M., Peloso G.M., Won H.H., Do R., van Iperen E., Kanoni S., Kruppa J., Mahajan A., Scott R.A., Willenberg C., Braund P.S., van Capelleveen J.C., Doney A.S., Donnelly L.A., Asselta R., Merlini P.A., Duga S., Marziliano N., Denny J.C., Shaffer C.M., El-Mokhtari N.E., Franke A., Gottesman O., Heilmann S., Hengstenberg C., Hoffman P., Holmen O.L., Hveem K., Jansson J.H., Jöckel K.H., Kessler T., Kriebel J., Laugwitz K.L., Marouli E., Martinelli N., McCarthy M.I., Van Zuydam N.R., Meisinger C., Esko T., Mihailov E., Escher S.A., Alver M., Moebus S., Morris A.D., Müller-Nurasyid M., Nikpay M., Olivieri O., Lemieux Perreault L.P., AlQarawi A., Robertson N.R., Akinsanya K.O., Reilly D.F., Vogt T.F., Yin W., Asselbergs F.W., Kooperberg C., Jackson R.D., Stahl E., Strauch K., Varga T.V., Waldenberger M., Zeng L., Kraja A.T., Liu C., Ehret G.B., Newton-Cheh C., Chasman D.I., Chowdhury R., Ferrario M., Ford I., Jukema J.W., Kee F., Kuulasmaa K., Nordestgaard B.G., Perola M., Saleheen D., Sattar N., Surendran P., Tregouet D., Young R., Howson J.M., Butterworth A.S., Danesh J., Ardissino D., Bottinger E.P., Erbel R., Franks P.W., Girelli D., Hall A.S., Hovingh G.K., Kastrati A., Lieb W., Meitinger T., Kraus W.E., Shah S.H., McPherson R., Orho-Melander M., Melander O., Metspalu A., Palmer C.N., Peters A., Rader D., Reilly M.P., Loos R.J., Reiner A.P., Roden D.M., Tardif J.C., Thompson J.R., Wareham N.J., Watkins H., Willer C.J., Kathiresan S., Deloukas P., Samani N.J., Schunkert H. (2016). Coding variation in ANGPTL4, LPL, and SVEP1 and the risk of coronary disease. N Engl J Med.

[6] Jung I.H., Elenbaas J.S., Alisio A., Santana K., Young E.P., Kang C.J., Kachroo P., Lavine K.J., Razani B., Mecham R.P., Stitziel N.O. (2021). SVEP1 is a human coronary artery disease locus that promotes atherosclerosis. Sci Transl Med.

[7] Sato-Nishiuchi R., Nakano I., Ozawa A., Sato Y., Takeichi M., Kiyozumi D., Yamazaki K., Yasunaga T., Futaki S., Sekiguchi K. (2012). Polydom/SVEP1 is a ligand for integrin α9β1. J Biol Chem.

[8] Hynes R.O. (2002). Integrins: bidirectional, allosteric signaling machines. Cell.

[9] Stupack D.G., Cheresh D.A. (2002). Get a ligand, get a life: integrins, signaling and cell survival. J Cell Sci.

[10] Modvig S., Jeyakumar J., Marquart H.V., Christensen C. (2023). Integrins and the metastasis-like dissemination of acute lymphoblastic leukemia to the central nervous system. Cancers (Basel).

[11] Bazigou E., Xie S., Chen C., Weston A., Miura N., Sorokin L., Adams R., Muro A.F., Sheppard D., Makinen T. (2009). Integrin-alpha9 is required for fibronectin matrix assembly during lymphatic valve morphogenesis. Dev Cell.

[12] Jain M., Dev R., Doddapattar P., Kon S., Dhanesha N., Chauhan A.K. (2021). Integrin α9 regulates smooth muscle cell phenotype switching and vascular remodeling. JCI Insight.

[13] Huang B., Niu Y., Chen Z., Yang Y., Wang X. (2020). Integrin α9 is involved in the pathopoiesis of acute aortic dissection via mediating phenotype switch of vascular smooth muscle cell. Biochem Biophys Res Commun.

[14] Jain M., Chauhan A.K. (2022). Role of integrins in modulating smooth muscle cell plasticity and vascular remodeling: from expression to therapeutic implications. Cells.

[15] Morris G.E., Denniff M.J., Karamanavi E., Andrews S.A., Kostogrys R.B., Bountziouka V., Ghaderi-Najafabadi M., Shamkhi N., McConnell G., Kaiser M.A., Carleton L., Schofield C., Kessler T., Rainbow R.D., Samani N.J., Webb T.R. (2022). The integrin ligand SVEP1 regulates GPCR-mediated vasoconstriction via integrins α9β1 and α4β1. Br J Pharmacol.

[16] Elenbaas J.S., Pudupakkam U., Ashworth K.J., Kang C.J., Patel V., Santana K., Jung I.H., Lee P.C., Burks K.H., Amrute J.M., Mecham R.P., Halabi C.M., Alisio A., Di Paola J., Stitziel N.O. (2023). SVEP1 is an endogenous ligand for the orphan receptor PEAR1. Nat Commun.

[17] Patel R.B., Dhanesha N., Sutariya B., Ghatge M., Doddapattar P., Barbhuyan T., Kumskova M., Leira E.C., Chauhan A.K. (2023). Targeting neutrophil α9 improves functional outcomes after stroke in mice with obesity-induced hyperglycemia. Stroke.

[18] Gupta S.K., Oommen S., Aubry M.C., Williams B.P., Vlahakis N.E. (2013). Integrin α9β1 promotes malignant tumor growth and metastasis by potentiating epithelial-mesenchymal transition. Oncogene.

[19] Masià A., Almazán-Moga A., Velasco P., Reventós J., Torán N., Sánchez de Toledo J., Roma J., Gallego S. (2012). Notch-mediated induction of N-cadherin and α9-integrin confers higher invasive phenotype on rhabdomyosarcoma cells. Br J Cancer.

[20] Zhang J., Na S., Liu C., Pan S., Cai J., Qiu J. (2016). MicroRNA-125b suppresses the epithelial-mesenchymal transition and cell invasion by targeting ITGA9 in melanoma. Tumour Biol.

[21] Jung I.H., Elenbaas J.S., Burks K.H., Amrute J.M., Xiangyu Z., Alisio A., Stitziel N.O. (2022). Vascular smooth muscle- and myeloid cell-derived integrin α9β1 does not directly mediate the development of atherosclerosis in mice. Atherosclerosis.

[22] Palmer E.L., Rüegg C., Ferrando R., Pytela R., Sheppard D. (1993). Sequence and tissue distribution of the integrin alpha 9 subunit, a novel partner of beta 1 that is widely distributed in epithelia and muscle. J Cell Biol.

[23] Singh P., Reimer C.L., Peters J.H., Stepp M.A., Hynes R.O., Van De Water L. (2004). The spatial and temporal expression patterns of integrin alpha9beta1 and one of its ligands, the EIIIA segment of fibronectin, in cutaneous wound healing. J Invest Dermatol.

[24] Bloom S.I., Islam M.T., Lesniewski L.A., Donato A.J. (2023). Mechanisms and consequences of endothelial cell senescence. Nat Rev Cardiol.

[25] Souilhol C., Serbanovic-Canic J., Fragiadaki M., Chico T.J., Ridger V., Roddie H., Evans P.C. (2020). Endothelial responses to shear stress in atherosclerosis: a novel role for developmental genes. Nat Rev Cardiol.

[26] Xu Q. (2006). The impact of progenitor cells in atherosclerosis. Nat Clin Pract Cardiovasc Med.

[27] Singh P., Chen C., Pal-Ghosh S., Stepp M.A., Sheppard D., Van De Water L. (2009). Loss of integrin alpha9beta1 results in defects in proliferation, causing poor re-epithelialization during cutaneous wound healing. J Invest Dermatol.

[28] Huang X.Z., Wu J.F., Ferrando R., Lee J.H., Wang Y.L., Farese R.V., Sheppard D. (2000). Fatal bilateral chylothorax in mice lacking the integrin alpha9beta1. Mol Cell Biol.

[29] Bennett M.R., Sinha S., Owens G.K. (2016). Vascular smooth muscle cells in atherosclerosis. Circ Res.

[30] Johnson J.L. (2014). Emerging regulators of vascular smooth muscle cell function in the development and progression of atherosclerosis. Cardiovasc Res.

[31] Rekhter M.D., Gordon D. (1995). Active proliferation of different cell types, including lymphocytes, in human atherosclerotic plaques. Am J Pathol.

[32] Morooka N., Futaki S., Sato-Nishiuchi R., Nishino M., Totani Y., Shimono C., Nakano I., Nakajima H., Mochizuki N., Sekiguchi K. (2017). Polydom is an extracellular matrix protein involved in lymphatic vessel remodeling. Circ Res.

[33] Thurston G., Daly C. (2012). The complex role of angiopoietin-2 in the angiopoietin-tie signaling pathway. Cold Spring Harb Perspect Med.

[34] Fiedler U., Reiss Y., Scharpfenecker M., Grunow V., Koidl S., Thurston G., Gale N.W., Witzenrath M., Rosseau S., Suttorp N., Sobke A., Herrmann M., Preissner K.T., Vajkoczy P., Augustin H.G. (2006). Angiopoietin-2 sensitizes endothelial cells to TNF-alpha and has a crucial role in the induction of inflammation. Nat Med.

[35] Anisimov A., Fang S., Hemanthakumar K.A., Örd T., van Avondt K., Chevre R., Toropainen A., Singha P., Gilani H., Nguyen S.D., Karaman S., Korhonen E.A., Adams R.H., Augustin H.G., Öörni K., Soehnlein O., Kaikkonen M.U., Alitalo K. (2023). The angiopoietin receptor Tie2 is atheroprotective in arterial endothelium. Nat Cardiovasc Res.

[36] Nanda N., Bao M., Lin H., Clauser K., Komuves L., Quertermous T., Conley P.B., Phillips D.R., Hart M.J. (2005). Platelet endothelial aggregation receptor 1 (PEAR1), a novel epidermal growth factor repeat-containing transmembrane receptor, participates in platelet contact-induced activation. J Biol Chem.

[37] Kauskot A., Di Michele M., Loyen S., Freson K., Verhamme P., Hoylaerts M.F. (2012). A novel mechanism of sustained platelet αIIbβ3 activation via PEAR1. Blood.

[38] Johnson A.D., Yanek L.R., Chen M.H., Faraday N., Larson M.G., Tofler G., Lin S.J., Kraja A.T., Province M.A., Yang Q., Becker D.M., O'Donnell C.J., Becker L.C. (2010). Genome-wide meta-analyses identifies seven loci associated with platelet aggregation in response to agonists. Nat Genet.

[39] Keramati A.R., Chen M.H., Rodriguez B.A.T., Yanek L.R., Bhan A., Gaynor B.J., Ryan K., Brody J.A., Zhong X., Wei Q., Kammers K., Kanchan K., Iyer K., Kowalski M.H., Pitsillides A.N., Cupples L.A., Li B., Schlaeger T.M., Shuldiner A.R., O'Connell J.R., Ruczinski I., Mitchell B.D., Faraday N., Taub M.A., Becker L.C., Lewis J.P., Mathias R.A., Johnson A.D., NHLBI Trans-Omics for Precision (TOPMed) Consortium (2021). Genome sequencing unveils a regulatory landscape of platelet reactivity. Nat Commun.

[40] Ansari N., Najafi S., Shahrabi S., Saki N. (2021). PEAR1 polymorphisms as a prognostic factor in hemostasis and cardiovascular diseases. J Thromb Thrombolysis.

[41] Lewis J.P., Riaz M., Xie S., Polekhina G., Wolfe R., Nelson M., Tonkin A.M., Reid C.M., Murray A.M., McNeil J.J., Shuldiner A.R., Lacaze P. (2020). Genetic variation in PEAR1, cardiovascular outcomes and effects of aspirin in a healthy elderly population. Clin Pharmacol Ther.

[42] Xu K., Ye S., Zhang S., Yang M., Zhu T., Kong D., Chen J., Xu L., Li J., Zhu H., Wang F., Yang L., Zhang J., Fan Y., Ying L., Hu X., Zhang X., Chan N.C., Li C. (2019). Impact of platelet endothelial aggregation receptor-1 genotypes on platelet reactivity and early cardiovascular outcomes in patients undergoing percutaneous coronary intervention and treated with aspirin and clopidogrel. Circ Cardiovasc Interv.

