# Supplementary material for: Integrin α9β1 deficiency does not impact the development of atherosclerosis in mice
Source: Heliyon. 2024 Feb 9;10(4):e25760. doi: 10.1016/j.heliyon.2024.e25760 (PMC10869861; doi:10.1016/j.heliyon.2024.e25760)
Supplement: Multimedia component 1 [file mmc1.pdf]

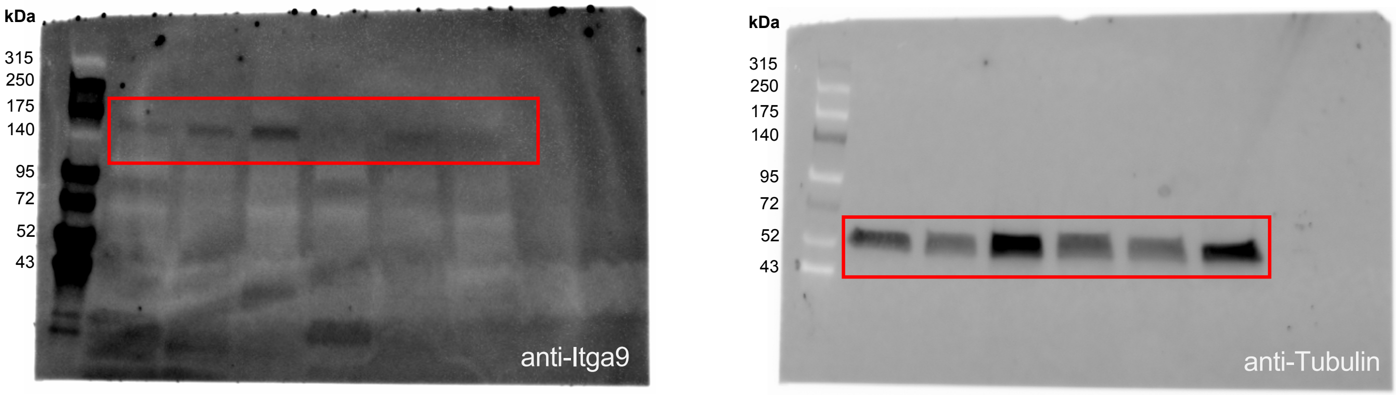

Supplementary Figure 1. Uncropped images of immunoblots presented in Figure 1B. Red boxes depict cropped areas shown in Figure 1B.
